# Well siblings’ experiences of living with a child following a traumatic brain injury: a systematic review protocol

**DOI:** 10.1186/s13643-019-1005-9

**Published:** 2019-04-02

**Authors:** Katie Hill, Maria Brenner

**Affiliations:** School of Nursing and Midwifery, Trinity College Dublin, Dublin, Ireland

**Keywords:** Traumatic brain injury, Siblings, Children, Family, Experiences

## Abstract

**Background:**

The aim of this systematic review is to synthesize the available evidence identified through a systematic search on well siblings’ experiences of living with a child following a traumatic brain injury (TBI). Brain injuries in children have been referred to as the “silent epidemic” of current times. Brain injuries in children are also recognized as a global public health concern, with the impact on children, effects on family life, and caregiving markedly misunderstood and underestimated. It is widely recognized that a serious brain injury impacts on the whole family, both immediate and extended regardless of the age of the individual who experiences the brain injury. While some research refers to parental experiences of children with TBIs and caregivers experiences, there is a dearth of literature relating to the impact on well siblings and their perspectives. Well siblings’ experiences regarding the impact of living with a child post-TBI are not well understood. In order to advance the delivery of family nursing care in the home, an understanding of the well siblings’ experiences is fundamental.

**Methods:**

The search will be conducted using seven medical and healthcare databases for articles published up until February 2019. Two reviewers will independently screen the articles for inclusion and assess for study quality using the standardized critical appraisal instrument from the Joanna Briggs Institute Qualitative Assessment and Review Instrument (JBI-QARI). Two reviewers will extract data from each study and carry out data analysis to uncover themes within the literature. Data synthesis of findings will be carried out using JBI-QARI.

**Discussion:**

It is anticipated that the findings of the proposed review will be of interest to health and social care professionals, particularly those working in units where children have suffered TBIs, their well siblings, and families. The aim is to identify well siblings’ experiences which can inform enhanced care delivery to the families of children following a TBI. The findings of this review will provide evidence to aid professionals with the assessment of siblings’ needs to enhance their sense of self within the family unit. Future directions, in addition to potential limitations of the approach, will be discussed.

**Systematic review registration:**

PROSPERO 2018 CRD42018111036

**Electronic supplementary material:**

The online version of this article (10.1186/s13643-019-1005-9) contains supplementary material, which is available to authorized users.

## Background

The aim of this review protocol is to outline the stages required to synthesize the available evidence found through a systematic search, on well siblings’ experiences of living with a child following a traumatic brain injury (TBI). The systematic review will systematically search for, appraise, and synthesize the available evidence found on these experiences. Following this, themes will be collated to form suggestions for how best to meet the needs of these children and subsequently provide an overall improved care provision for them. No systematic review surrounding this topic has been undertaken previously.

Brain injuries in children have been referred to in the literature as the “silent epidemic” of current times, with the impact on children, effects on family life, and caregiving markedly misunderstood and underestimated [1, 2]. It is widely recognized that a serious brain injury impacts on the whole family, both immediate and extended, regardless of the age of the individual who experiences the brain injury [3, 4].

TBI is defined as a type of non-degenerative acquired brain injury as a result of an external impact or insult to the brain including a blow, bump, or a penetrating head injury that disrupts normal brain functioning, as a result of a cognitive nature, degenerative conditions, and birth injuries [5]. There are a number of causes of TBI in children including falls, motor vehicle accidents, and assaults. As a result of a TBI, there can be some degree of physical, intellectual, or psychosocial disability, depending on the severity on the injury ranging from “mild” noted as a brief change in mental status or consciousness to “severe,” an extended period of unconsciousness after the injury, which can adversely affect a child’s social well-being or educational ability [6–9]. Physical changes may include changes in bowel and bladder function, changes in level of consciousness, impaired movement and coordination, reduced muscle strength, and onset of seizures [10]. Other behavioral changes associated with TBI include agitation, anxiety, irritability, changes in sleep patterns, and decreased social functioning leading to challenges in school, which can impact on wider family life and not just the child with TBI [11–13]. These changes do not always present themselves immediately after the injury but may become evident as the child continues to grow and present as developmental delays affecting social relationships and overall quality of life [1, 9, 11, 14–19]. As a result, families can experience a sense of loss and stress due to the significant alterations in their lives and become overwhelmed with the magnitude of medical complexity while trying to maintain family life [20, 21].

TBI in children comes under the growing phenomenon of complex care, because of these symptoms changing over time [7, 22]. Brenner et al. [23] highlighted that complex care needs in children are dynamic and continuing over time, with TBI firmly situated within this area. The challenge in caring for these children and their families is to provide the optimum care in a potentially every changing situation and throughout the trajectory of illness, from the time of diagnosis across many weeks, months, and years.

TBI is a global public health concern with over 3 million children estimated to be affected annually [24]. Incidence rates vary across the globe, with most countries reporting a range between 47 and 280 per 100,000 children [24], with falls and motor vehicle accidents reported as the most common causes. Numerous reports from various countries exist, although a general epidemiologic overview of TBI relating to the global pediatric population is lacking. Studies from Asia indicate that TBI contributes to more than half of pediatric injuries in Iran, approximately 20% of emergency department admissions in India, and around 30% of pediatric injuries in Korea [25–27]. In the USA, it is estimated that, annually, more than one million children have a TBI, with 30,000 sustaining injuries that result in lifelong impairments and they are a leading cause of death and disabilities in children [5]. In New Zealand, the average annual incidence of TBIs between age 0–25 years was 1.10–2.36 per 100 [28]. Other studies have yielded similar results with a higher incidence observed in the adolescent and young adult group due to increased risk-taking behavior in this group including motor vehicle activities [29]. In England, an approximate annual incidence of hospital events were recorded for children with a head injury as 400 per 100,000 children under the age of 15 between 2012 and 2013 [30]. While these figures are predominantly estimates, it gives an insight into the significance and extent of TBI in children.

Sibling relationships are significant from a very early age, regardless of family systems or dynamics [31, 32]. Their relationships are complex and diverse, with children themselves actively shaping these relationships. Siblings play a fundamental role in children’s lives and when those children sustain a traumatic brain injury siblings are at the forefront and also experience life-changing events as a result. It is unknown what impact the evolving changes post-TBI may have on well siblings living with this child and how they cope with these changes in family life, supporting the need for further research in this area. There has been no published systematic review in the area conducted before, which strengthens the justification for this important piece of research.

Parents’ and caregivers’ experiences of caring for children following TBI’s have been explored in the literature [20, 33–37]. The increased levels from the burden of caregiving on parents and psychological symptoms including stress have been highlighted, which could potentially impact on siblings but this was not overtly specified [3, 38]. TBI affects the entire family, not just the child with the injury, with adjustment necessary from parents in order to function and manage their child, while experiencing emotional distress and relationship dissonance which added to the challenges of their parenting roles [35]. In families where the child with TBI is not an only child, the impact on the well siblings cannot be underestimated. The impact on these well siblings of living with a child following a TBI is significant for their own health, wellbeing, and development and is an area that requires investigation.

Children with complex care need influence the family and well siblings in a number of ways, including siblings’ bond, parent-child dynamic and relationships, rivalry between siblings, relationships between siblings, and ultimately, a loss of normal life [39–41]. Siblings’ experiences of living with children with other complex care needs and chronic conditions have been examined in previous research studies, and the effects on well siblings were explored [39, 42–50]. Some findings reported on well siblings being positively impacted by their lived experiences, through the expression of positive social skills, increased empathy, and more caring personalities [51] with these well siblings actively participating in the child’s care [39]. Conversely, other findings included difficulties adjusting from the well sibling, including expressing negative behaviors such as anger, irritability, and aggression or through anxiety and social or emotional withdrawal [39, 46, 52]. However, the process of overall adjustment for well siblings of chronically ill children is inconclusive with both positive and negatives experiences reported on by the siblings in numerous studies [39, 44, 53]. While these studies relate to children with wider complex care needs, the principles may be mirrored in well siblings of children with TBI.

There are few research studies exploring siblings’ experiences of children with brain injuries [1, 54, 55]. Their findings included family members experiencing significant emotional disturbances, stress and frustration following injury, and difficulties with separations due to long hospitalizations leading to lack of contact with the child for well siblings, with parents feeling they neglected their healthier children. Well siblings of children that sustained a significant TBI are likely to be affected by family changes and functioning post-injury with a possible risk of developing psychological difficulties [55], but minimal research exists on sibling outcomes and responses.

Further research is crucial to fully understand well siblings’ experiences and responses for TBI. Areas to be explored will include experiences at the time of their well siblings’ injury or illness, experiences of the hospitalization period, and subsequent rehabilitation process, before returning home. Perceptions of life changes and roles within the family will also be explored. Understanding these experiences of well siblings living with a child post-TBI is needed to advance the delivery of family nursing care in the home.

## Methods

### Design

The reporting will follow the Preferred Reporting Items for Systematic Reviews and Meta-Analysis (PRISMA) framework (see Additional file 1).

### Aim of the review

The aim of the proposed review is to evaluate and synthesize the evidence identified through this systematic search of experiences of well siblings’ living with a child with a TBI and subsequently to highlight the quality of that evidence.

The review question will be: What are well siblings’ experiences of living with a child following a traumatic brain injury?

A systematic review is the chosen methodology for this study, to synthesize all of the available evidence identified through this systematic search, allowing analysis of the research and subsequently extracting the experiences of well siblings living with a child who has a TBI [56].

While research involving children with TBIs, including experiences of parents, families, and healthcare professionals, have been documented in the literature, the aim of this review is to solely explore the well siblings’ experiences. Through this review, a deeper understanding of well siblings’ experiences will be presented to improve their involvement and to promote family-centered care delivery when a child has a TBI.

### Participants

The aim of having pre-specified inclusion and exclusion criteria is to reduce or eliminate bias to ensure that outcomes cannot be adjusted to fit the proposed research question. The PEOT framework for qualitative analysis is appropriate for this review and identifies the population, exposure, outcomes, and types of studies [57]. For studies to be included in this review, they must report on:

### Population

1. Well siblings who have experience of living with a child following a TBI will be included as they are the focus of this review.
  Siblings who have experience of living with a child with a brain injury as a result of birth trauma or an injury of a congenital nature will be excluded based on the previously explained definition of a TBI.
  Any other family members, for example, parents or grandparents, will be excluded from this study. Patients’ views will not be included in this study as the focus is on the siblings, not the child themselves. Health care professionals will not be included in this study.

2. Within the literature, siblings must be identified to be under the age of 18. Anyone over 18 years old will not be included as children are classified as individuals under 18 years of age [58].
  Where studies report on the experience of siblings outside of this age range, they will not be included.

### Exposure

The types of exposure for inclusion are any discussions in the literature relating to areas where children with TBIs and their well siblings may be cared for. The locations will include rehabilitation centers, neurology wards, family care programs, and community care settings.

### Outcomes

The outcome of interest in this review is to explore well siblings’ experiences of living with a child following a TBI. The experiences of these well siblings are necessary to understand the impact on family life and are crucial to further care delivery. In order to create policies for caring for well siblings during the time of a TBI, these need to be investigated.

All references to thoughts, experiences, feelings, views, opinions, perceptions, beliefs, and actions relating to the topic will be included.

### Types of studies

In order to best answer the review question relating to experiences and views, qualitative studies which explore well siblings’ experiences on living with a child following a TBI will be the focus for inclusion. All primary qualitative research studies, exploring well siblings’ experiences, published up until February 2019 will be included, as this is the time when the study selection will be completed. Quantitative studies, which offer no qualitative data on well siblings’ experiences, will be excluded. Mixed methods studies, offering both qualitative and quantitative data, will be examined for their qualitative data only. Non-research studies including editorials will be excluded in the review but will be consulted for the wider background and cultural context of the review.

### Search strategy

The search strategy regulates the quality of the literature search and will develop a comprehensive list of all relevant studies for the chosen topic [57]. All relevant studies relating to the review question must be retrieved to provide validity and ensure the widest possible search is undertaken. An exhaustive search of the literature will establish what is known and unknown in the area [59]. There will be no language restriction placed on the search. There will be no time limit placed on the search as older literature may provide fundamental information around this area. The search strategy is clearly documented so the reader can assess the rigor, integrity, and repeatability of the study.

### Search terms

Using the PEO format, identifying population, exposure, and outcomes, synonyms for each term were derived using a thesaurus. A wide range of terms will be used to ensure no relevant article would be overlooked (see Additional file 2). In some cases, TBI and spinal cord injury can be interlinked following a traumatic event [60]. Subsequently, this was also used as a keyword to ensure no relevant studies would be missed. The writer will develop a search string/strings and Boolean operators AND/OR will be used to filter the research to source relevant material.

### Data sources

Using the keywords identified in the search strategy strings, an in-depth search of all relevant databases will be completed [61]. Medical, healthcare, and social science databases will be the focus for the searches including CINAHL (Cumulative Index to Nursing and Allied Health Literature) Complete (providing full text dating back to 1937), MEDLINE (inception date 1879), PsycINFO (inception date 1967), PsycARTICLES (inception date 1894), EMBASE (Excerpta Medica Database) (inception date 1947), AMED (Allied and Complementary Medicine) (inception date 1980), and Social Sciences Full Text (formerly H. W. Wilson) (inception date 1983). Each database has been chosen for its relevance to the subject area and to ensure a wide range of literature will be explored: CINAHL as it is the most comprehensive source of nursing and allied health journals, MEDLINE as it is a database of life sciences and biomedical information, PsycINFO and PsycARTICLES for their behavioral and social science research elements, EMBASE as it is the most comprehensive biomedical literature database, AMED for their approach to complementary medicine and alternative treatments, and Social Sciences Full Text as it focuses on the scientific studies of human society and social relationships. Grey literature, in the form of unpublished research evidence, using the webpages opengrey and greylit, will be explored to ensure a comprehensive search is completed [62]. The searches and results obtained will be recorded and saved electronically. The PRESS initiative [63] will be used as a checklist for peer review of the search strategies (see Additional file 3).

### Study selection

The titles and abstracts of the articles which will be retrieved using the search strategy outlined above and will be scanned independently by two reviewers to ensure transparency. The articles, which are chosen for inclusion in the review, must meet the pre-defined inclusion and exclusion criteria. After the screening process is completed, the papers which have been identified as meeting the criteria will be sourced in full text and reviewed by both reviewers. This independent double-checking process is to ensure all of the papers are applicable to the review question and meet the pre-defined inclusion criteria [64]. If there is a disagreement regarding the selection and inclusion of a paper, a third researcher with expertise in the area will be consulted if necessary, to determine inclusion or exclusion of the study [64]. The reasons for exclusions of studies in their review will be documented.

### Quality assessment

Quality assessment will be conducted to assess the value of the research, assess the quality of the study, and avoid drawing inaccurate conclusions [65]. Quality assessment will only be completed when studies are selected and agreed on by both reviewers for inclusion. The quality assessment identifies studies strengths and weaknesses and is one of the key components that distinguishes a systematic review from a narrative review along with the systematicity and transparency of choices made throughout the process [66]. The quality of each study selected for inclusion will be appraised using the standardized critical appraisal instruments from the Joanna Briggs Institute Qualitative and Review Instrument (JBI-QARI). This appraisal tool includes ten criteria relating to qualitative research design, philosophy, and trustworthiness.

This process will be independently assessed by the two reviewers to eliminate subjective bias and to ensure equity across quality assessment scores [56]. Depending on the outcomes of the quality assessment of the studies and the number of studies initially selected for the review, studies may be excluded if they are deemed poor quality based on the results. If studies are excluded based on poor credibility, it will strengthen the findings of the review. However, this will not be decided until the final number of studies is compiled. If studies with low credibility scores are to be included due to a limited number of studies in the review, this will be highlighted as a limitation by the reviewer.

### Data extraction

Data extraction must be accumulated in a transparent manner in a review. The two reviewers will independently extract the data using the standardized data extraction tool from JBI-QARI. If there is more than one publication reporting data from the same study, the first publication will be chosen to avoid double reporting data on the same participants. However, both publications will be reviewed thoroughly, by both reviewers, in case the second paper reports additional or more in-depth relevant findings for inclusion in the review. Findings will be rated by their degree of credibility according to the JBI-QARI three levels of credibility: unequivocal, credible, and unsupported [67].

### Data analysis/synthesis

Qualitative research findings will be merged using JBI-QARI. This will involve the synthesis of findings in order to generate a set of statements which will represent the collective [68]. These findings will be rated according to their quality and categorized based on their similarity of meaning [68]. These categories will then be subjected to a metasynthesis to generate a comprehensive set of synthesized findings which can provide a basis for evidence-based practice [67]. Both reviewers will be involved in the process of data analysis and synthesis, through the process of independent synthesis to ensure that the themes identified are accurate.

## Discussion

It is anticipated that the findings of the proposed review will be of interest to health and social care professionals, particularly those working in units where children have suffered TBIs, their well siblings, and families. Examples of these units could be respite units, rehabilitation settings, neurology wards in children’s hospitals, or neurology wards in adult hospitals where children may be cared for. Currently, services provided for siblings of children with TBI are limited and appear to be ad hoc depending on the availability of healthcare professionals working within a service, for example, play therapists or child psychologists. It is likely that the findings will identify well siblings’ experiences which can improve care delivery to this cohort and may support the need for healthcare professionals to consider siblings as fundamental participants affecting the ill child and families’ well-being. It is predicted the findings of this study will make a valuable contribution to the existing body of knowledge in this area.

### Limitations

If it is necessary to implement restrictions regarding the English language after the search, due to restrictions with translation availability, this will be recognized as a limitation in the final review.

### Dissemination

The findings of the review will be disseminated through publication in peer review journals and presentation at relevant conferences and educational events. It is likely these findings will be of interest to health and social care professionals caring for children with a TBI and to policy makers to contribute to further care planning and service development.

## Conclusion

This protocol aimed to justify the rationale of conducting a review of well siblings’ experiences of living with a child who has suffered a TBI by providing the background to this topic including literature surrounding the current situation. The review question was highlighted and the proposed methodology for undertaking the review was outlined. Understanding experiences is paramount for envisioning behaviors, which can inform practice, develop policy, and lead to further research.

## Additional files

Additional file 1: PRISMA Checklist. (DOCX 21 kb)

Additional file 2: Search String. (DOCX 17 kb)

Additional file 3: PRESS initiative [62]. (DOCX 14 kb)

## Abbreviations

AMED: Allied and Complementary Medicine
CINAHL: Cumulative Index to Nursing and Allied Health Literature
EMBASE: Excerpta Medica Database
MEDLINE: Medical Literature Analysis and Retrieval System Online
PRISMA: Preferred Reporting Items for Systematic Reviews and Meta-Analysis
PsycARTICLES: Scientific journal to inform meaningful research
PsycINFO: Psychology Information Database
TBI: Traumatic brain injury
