# Supplementary material for: Well siblings’ experiences of living with a child following a traumatic brain injury: a systematic review protocol
Source: Syst Rev. 2019 Apr 2;8:81. doi: 10.1186/s13643-019-1005-9 (PMC6446255; doi:10.1186/s13643-019-1005-9)
Supplement: Supplementary file 2 — Search String. (DOCX 17 kb) [file 13643_2019_1005_MOESM2_ESM.docx]

| Terms combined with | Population AND | Exposure AND | Outcomes AND |
| --- | --- | --- | --- |
| OR | 1. Sibling (s) | 25. Neurology ward | 38. Actions |
| OR | 2. Child (ren) | 26. Children’s ward | 39. Experiences |
| OR | 3. Brother (s) | 27. Children’s hospital | 40. Understanding |
| OR | 4. Sister (s) | 28. Rehabilitation centre | 41. Belief |
| OR | 5. Young Person (s) | 29. Rehabilitation facility | 42. Practice |
| OR | 6. Relative (s) | 30. Community | 43. Reality |
| OR | 7. Family | 31. Respite unit | 44. Sense |
| OR | 8. Pediatric | 32. Long term care setting | 45. Struggle |
| OR | 9. Paediatric | 33. Disability unit | 46. Wisdom |
| OR | 10. Traumatic brain injury or TBI or tbi | 34. Emergency room or ER | 47. Thoughts |
| OR | 11. Acquired brain injury or ABI or abi | 35. Emergency department or ED | 48. Evidence |
| OR | 12. Brain injury | 36. Accident & emergency | 49. Exposure |
| OR | 13. Head trauma |  | 50. Efforts |
| OR | 14. Head injury |  | 51. Wishes |
| OR | 15. Spinal cord injury |  | 52. Desires |
| OR | 16. Traumatic Injury |  | 53. Perspectives |
| OR | 17. Trauma |  | 54. Preferences |
| OR | 18. trauma victim |  | 55. Needs |
| OR | 19. accident (s) |  | 56. Participation |
| OR | 20. injury |  | 57. Observation |
| OR | 21. injuries |  | 58. Involvement |
|  | 22. Combine 1 – 9 using OR | 37. Combine 25 - 36 using OR | 59. Combine 38 – 58 using OR |
|  | 23. Combine 10 - 21 using OR |  |  |
|  | 24. Combine 22 & 23 using AND |  | *Combine 24 & 37 & 59 using AND* |

**Additional File 2: Search String**
