# Supplementary material for: Well siblings’ experiences of living with a child following a traumatic brain injury: a systematic review protocol
Source: Syst Rev. 2019 Apr 2;8:81. doi: 10.1186/s13643-019-1005-9 (PMC6446255; doi:10.1186/s13643-019-1005-9)
Supplement: Supplementary file 3 — PRESS initiative [62]. (DOCX 14 kb) [file 13643_2019_1005_MOESM3_ESM.docx]

**Additional File 3: PRESS initiative [60]**

1. Assess whether the research question has been correctly translated into search concepts
2. Assess whether the elements addressing the search question have been correctly combined with Boolean and/or proximity operators
3. Assess all spelling and system syntax
4. Assess for correct search implementation by checking each line number and combination of numbers
5. Assess whether the search strategy was correctly adapted for each database used
6. Assess whether there is enough scope in the selection of subject headings to optimize recall
7. Assess whether the search terms without adequate subject heading coverage are well represented by free-text terms and whether additional synonyms and related terms are needed
8. Assess the adequate use of both subject headings and free-text terms used in combination
9. Assess whether all relevant spelling variants are covered by the search terms
10. Assess the relevance of the search terms to the search question
11. Assess whether the limits (including filters) use were appropriate and have been applied correctly
